# Association of pulse pressure and aortic root diameter in elderly Chinese patients with chronic heart failure

**DOI:** 10.3389/fcvm.2024.1366282

**Published:** 2024-03-01

**Authors:** Lu Chen, Wenhui Xie, Xuhui Hong, Huashan Hong

**Affiliations:** Department of Geriatrics, Fujian Key Laboratory of Vascular Aging, Fujian Institute of Geriatrics, Fujian Medical University Union Hospital, Fuzhou, Fujian, China

**Keywords:** pulse pressure, aortic root diameter, heart failure, arterial stiffness, elderly

## Abstract

**Background:**

High pulse pressure (PP) and aortic root diameter (AoD) are hallmarks of arterial stiffness or vascular aging and they are considered as risk factors for age-related cardiovascular disease, including heart failure (HF). However, the relationship between PP and AoD in patients with heart failure (HF) is uncertain. This study aimed to evaluate the relationship between PP and AoD in the middle-aged and the elderly with HF.

**Methods:**

A total of 1,027 Chinese middle-aged and elderly patients with HF, including HF with reduced ejection fraction (HFrEF), HF with mid-range EF (HFmrEF), and HF with preserved EF (HFpEF) were included in this study. Pearson correlation analysis was used to evaluate the relationship between PP and AoD in the three types of HF. Multiple linear regression analysis was performed to assess the factors that affected AoD. Multivariate logistic regression was performed to determine the association between the PP level quartiles and AoD. The results were validated in an independent dataset included a total of 378 consecutive patients with HFrEF hospitalized at the Pingtan Branch of Fujian Medical University Union Hospital (Fujian, China).

**Results:**

There was a positive correlation between PP and AoD in the middle-aged and the elderly with HFrEF. Multiple linear regression analysis revealed that PP, age, and body mass index (BMI) were independently correlated with AoD in HFrEF patients. In multivariate logistic regression analysis, an increased risk of aortic root dilation was observed in the highest quartile of the PP level compared with the lowest quartile. Age significantly interacted with PP (*p* = 0.047). A significant association between PP levels and AoD was only observed in patients ≥ 65 years old, but not in patients < 65 years old. In the validation dataset, PP was independently related to AoD in patients with HFrEF (*β* = 0.205, *p* = 0.001).

**Conclusions:**

PP level was independently and positively associated with AoD, especially in the elderly with HFrEF, but not in patients with HFmrEF and HFpEF. Arterial stiffening or vascular aging may play a certain role in the elderly HFrEF patients.

## 1 Introduction

Millions of adults worldwide suffer from heart failure (HF), which is associated with higher mortality rates, morbidity, and healthcare costs in the world (1, 2). Clinically, three types of HF are recognized: HF with reduced ejection fraction (HFrEF), HF with mid-range ejection fraction (HFmrEF), and HF with preserved ejection fraction (HFpEF) (3). Previous studies demonstrated that pulse pressure (PP) could be used to predict left ventricular hypertrophy and cardiovascular events (4–9). Wei et al. found that high PP predicted all-cause death, nonfatal myocardial infarction, nonfatal stroke, or hospitalization in patients with HF (10).

Several factors influence aortic root dilation, including age, gender, height, weight, body surface area (BSA), body mass index (BMI), and diseases such as hypertension, valvular heart disease, congenital heart disease, cardiomyopathy and ischemic cardiomyopathy (11). Recently, we and others have found that aortic root dilation also is one of the hall-mark of vascular aging or arterial stiffness (12, 13) which can lead to isolated systolic hypertension (14–16) and result in left ventricular remodeling, dysfunction, and heart failure (17, 18). A community-based cohort of Afro-American study found that aortic root diameter (AoD) was associated with an increased risk of cardiovascular events (19). The Framingham Heart Study indicated that the risk of incident HF increased with greater AoD at baseline and an increase in AoD over 8 years (20). AoD may be useful as a predictor of cardiovascular events even in the absence of aneurysmatic alterasions (21).

The precise relationship between PP and AoD is debatable (22–25) and there has yet to be a study looking into the association between them in the three types of HF. Furthermore, the relationship between PP and AoD in the middle-aged and the elderly with HF remains to be elucidated. Therefore, we aimed to investigate the relationship between PP and AoD in patients with HF in the present study.

## 2 Methods

### 2.1 Patients

We investigated the medical records of 1027 consecutive HF patients (age ≥ 45 years old) were hospitalized at the Fujian Medical University Union Hospital (Fujian, China) between January 2015 and December 2018. The patients' medical histories and relevant clinical characteristics were recorded. Patients were excluded from this study if they presented with any of the following conditions: acute myocardial infarction, acute myocarditis, significant valvular heart disease, congenital heart disease, renal failure [estimated glomerular filtration rate (eGFR) < 60 ml/min/1.73 m^2^], malignancy, and chronic inflammatory disease.

The validation dataset included a total of 378 consecutive patients with HFrEF hospitalized at the Pingtan Branch of Fujian Medical University Union Hospital (Fujian, China) between January 2015 and December 2018. The inclusion criteria included the HFrEF patients who underwent Echocardiography. The exclusion criteria included patients lacking echocardiography test, patients ≤ 45 years of age, and other confounding conditions, such as acute myocardial infarction, acute myocarditis, significant valvular heart disease, congenital heart disease, renal failure [estimated glomerular filtration rate (eGFR) < 60 ml/min/1.73 m^2^], malignancy, and chronic inflammatory disease.

### 2.2 Ethics statement

The study was conducted in accordance with the Declaration of Helsinki (as revised in 2013) and approved by the Medical Faculty of Fujian Medical University Union Hospital Ethics Committee (No. 2019KY004).

### 2.3 Anthropometric and hemodynamic variables

Clinical information, including age, BMI, white blood cell count, red blood cell count, blood lipid, serum creatinine, blood pressure, echocardiographic parameters, medical history of hypertension, diabetes and HF, and use of cardiovascular drugs, was extracted from medical records. BMI was calculated using the following formula: BMI (kg/m^2^) = weight (kg)/height^2^ (m^2^). Brachial BP determinations were performed in the supine position after a 15-min rest in the hospital by traditional mercury sphygmomanometry, with the first and the fifth Korotkoff sounds for SBP and DBP measurements, respectively. The average of the last 3 consecutive BP measurements was used for data analysis (26). PP was calculated using the following formula: PP = systolic blood pressure (SBP)—diastolic blood pressure (DBP). The eGFR was calculated using the Recommended equations for GFR estimation (27). The AoD was measured from the M-mode tracing as the maximal distance between the leading edge to the leading edge (L-L) convention of anterior and posterior aortic root wall at the maximal level of the sinuses of Valsalva, as recommended by the American Society of Echocardiography guidelines (28). Left ventricular dimensions were measured based on the American Society of Echocardiography recommendations. Aortic root dilation was defined as an AoD of ≥34 mm and ≥30 mm in males and females, respectively.

Patients were classified into non-smokers and current smokers (continuously smoking one or more cigarettes a day for at least six months) based on their smoking status (29). Hypertension was diagnosed as SBP ≥ 140 mmHg and/or DBP ≥ 90 mmHg and/or taking antihypertensive medications according to the European Society of Cardiology (ESC) guidelines (30).

### 2.4 Definition and type of HF

The diagnosis of HF was based on the symptoms or signs, electrocardiograms (ECG), chest radiographs, and echocardiography (31). HF was categorized as HFpEF if the left ventricular ejection fraction (LVEF) was ≥50%, HFmrEF if LVEF was 40%–49%, and HFrEF if LVEF was <40%. Chronic kidney disease (CKD) was diagnosed when eGFR was <90 ml/min/1.73 m^2^. Coronary heart disease (CHD) was defined as a history of coronary stent implantation or coronary artery bypass graft and myocardial ischemia on ECG, with symptoms typical of angina or myocardial infarction. Stroke was defined as the presence of a definite history of stroke or signs of cerebral infarction on computed tomography or magnetic resonance imaging.

### 2.5 Statistical analysis

The normality of the data was assessed by the Kolmogorov–Smirnov test. Normally distributed variables are presented as mean ± standard deviation and compared via student t test. Non-normally distributed variables are expressed as the median and interquartile range (IQR) and analyzed by Mann–Whitney *U* test. Categorical variables are expressed as numbers and percentages (%) and compared using the *χ*^2^ test or Fisher's exact test (if theoretical frequency T < 5). 1-way ANOVA followed by the Bonferroni post test for multiple comparisons. Linear correlations between variables were assessed using Pearson's correlation analysis. Non-linear associations between variables were assessed using restricted cubic spline analysis. Multiple linear regression analyses were performed to assess the independent determinants of AoD/BSA. The association between AoD/BSA and clinical parameters including patients' age, BMI, blood test indicators, hemodynamic parameters and echocardiographic indicators, was analyzed based on stepwise linear regression. This analysis was conducted by considering AoD/BSA as independent variables, and relevant clinical characteristics were set as dependent variables. Using the logistic regression analysis, PP was grouped by quartiles when analyzing the relationship between PP and aortic dilation risk using logistic regression analysis. The interactions between PP and aortic dilation risk were assessed by introducing a cross-product term into the regression models. Statistical significance was set at *p* < 0.05. All data were analyzed using the SPSS (version 19.0, IL, USA) software.

## 3 Results

### 3.1 Clinical characteristics of patients with HF

A total of 1,027 medical records of patients with HF were evaluated, including 270 (26.3%) HFrEF, 190 (18.5%) HFmrEF, and 567 (55.2%) HFpEF patients. The mean age of all patients was 69.4 ± 10.5 years, and 63.6% were males. The mean SBP and DBP were 133.1 ± 22.4 and 78.2 ± 12.9 mmHg, respectively, resulting in a mean PP of 54.8 ± 17.7 mmHg. Echocardiographic data were available for all patients; the AoD levels averaged 31.23 ± 4.16 mm. Of the 1,027 patients, 685 (66.7%) had a history of systemic hypertension, 360 (35.1%) had type 2 diabetes, and 623 patients (60.7%) had CHD. Among the HFrEF patients, 131 (48.5%) had a history of hypertension and 96 (35.6%) had type-2 diabetes. PP and AoD were 44.8 ± 14.0 mmHg and 31.36 ± 4.19 mm, 52.6 ± 16.5 mmHg and 40.00 ± 4.03 mm, and 60.3 ± 17.5 mmHg and 31.24 ± 4.18 mm in HFrEF, HFmrEF and HFpEF patients, respectively (Table 1).

**Table 1:** Comparison of clinical characteristics among patients with different types of HF.

Variables	All patients *n* = 1,027	HFrEF *n* = 270	HFmrEF *n* = 190	HFpEF *n* = 567	*p*
Age, years	69.4 ± 10.5	65.7 ± 10.3	67.8 ± 10.6	71.7 ± 9.9	<0.001
Male, *n* (%)	653 (63.6%)	201 (74.4%)	135 (71.1%)	317 (55.9%)	<0.001
BMI, kg/m^2^	24.19 ± 3.74	23.82 ± 3.79	24.08 ± 3.61	24.40 ± 3.76	0.096
SBP, mmHg	133.1 ± 22.4	121.6 ± 20.3	130.3 ± 21.6	139.4 ± 21.3	<0.001
DBP, mmHg	78.2 ± 12.9	76.8 ± 13.9	77.7 ± 13.7	79.1 ± 12.0	0.045
PP, mmHg	54.8 ± 17.7	44.8 ± 14.0	52.6 ± 16.5	60.3 ± 17.5	<0.001
AoD, mm	31.23 ± 4.16	31.36 ± 4.19	40.00 ± 4.03	31.24 ± 4.18	0.630
HR, bpm	76.6 ± 17.1	80.8 ± 18.8	80.1 ± 19.7	73.4 ± 14.5	<0.001
Past smoking, *n* (%)	570 (55.5%)	143 (53.0%)	101 (53.2%)	326 (57.5%)	0.361
Past drinking	629 (61.2%)	152 (56.3%)	98 (51.6%)	379 (66.8%)	<0.001
WBC, ×10^9^/L	7.11 ± 2.64	7.30 ± 2.80	8.03 ± 3.43	6.72 ± 2.13	<0.001
Hb, g/L	128.6 ± 19.1	133.7 ± 19.5	127.7 ± 19.1	127.1 ± 18.7	<0.001
HCT	39.1 ± 6.5	40.1 ± 5.8	38.4 ± 5.9	38.8 ± 6.9	0.006
TB, µmol/L	13.27 ± 7.96	15.90 ± 9.10	13.41 ± 8.47	11.97 ± 6.82	<0.001
ALB, g/L	36.97 ± 4.86	36.68 ± 4.82	36.81 ± 4.70	37.17 ± 4.93	0.354
ALT(IU/L) (log-transformed)	3.15 ± 0.70	3.33 ± 0.88	3.28 ± 0.66	3.02 ± 0.58	<0.001
AST(IU/L) (log-transformed)	3.30 ± 0.67	3.43 ± 0.78	3.54 ± 0.91	3.16 ± 0.46	<0.001
TG, mmol/L	1.49 ± 1.21	1.44 ± 1.22	1.44 ± 0.95	1.53 ± 1.29	0.560
TC, mmol/L	4.03 ± 1.53	3.99 ± 1.11	3.99 ± 1.03	4.06 ± 1.82	0.767
HDL-C, mmol/L	1.06 ± 0.34	1.00 ± 0.33	1.04 ± 0.29	1.09 ± 0.35	0.001
LDL-C, mmol/L	2.57 ± 0.99	2.59 ± 0.95	2.57 ± 0.93	2.56 ± 1.03	0.929
eGFR, mL/min/1.73 m^2^	83.0 ± 39.1	82.8 ± 58.4	83.9 ± 30.7	82.9 ± 28.8	0.979
UA, µmol/L	404.4 ± 126.8	457.6 ± 143.4	404.5 ± 111.6	378.9 ± 115.0	<0.001
FBG, mmol/L	6.11 ± 2.29	6.02 ± 2.24	6.44 ± 2.50	6.04 ± 2.23	0.082
NT-proBNP(pg/ml)					
(log-transformed)	6.88 ± 1.43	7.74 ± 1.24	7.35 ± 1.33	6.31 ± 1.28	<0.001
HbAlC, %	6.72 ± 1.39	6.80 ± 1.34	6.80 ± 1.46	6.65 ± 1.38	0.239
LVED, mm	53.56 ± 9.65	64.00 ± 9.46	53.91 ± 7.01	48.47 ± 5.63	<0.001
LAD, mm	41.48 ± 8.45	46.22 ± 8.97	41.11 ± 7.74	39.42 ± 7.59	<0.001
NYHA, *n* (%)					
I	21 (2.0%)	2 (0.7%)	10 (5.3%)	9 (1.6%)	0.002
II	597 (58.1%)	93 (34.4%)	96 (50.5%)	408 (72.0%)	<0.001
III	298 (29.0%)	115 (42.6%)	57 (30.0%)	126 (22.2%)	<0.001
IV	111 (10.8%)	60 (22.2%)	27 (14.2%)	24 (4.2%)	<0.001
Hypertension, *n* (%)	685 (66.7%)	131 (48.5%)	112 (58.9%)	442 (78.0%)	<0.001
DM (*n*, %)	360 (35.1%)	96 (35.6%)	72 (37.9%)	192 (33.9%)	0.590
CHD, *n* (%)	623 (60.7%)	126 (46.7%)	146 (76.8%)	351 (61.9%)	<0.001
DCM, *n* (%)	186 (18.1%)	132 (48.9%)	26 (13.7%)	28 (4.9%)	<0.001
Stroke, *n* (%)	116 (11.3%)	18 (6.7%)	19 (10.0%)	79 (13.9%)	0.007
CKD, *n* (%)	66 (6.4%)	27 (10.0%)	11 (5.8%)	28 (4.9%)	0.019
ACEI or ARB, *n* (%)	616 (60.0%)	165 (61.1%)	111 (58.4%)	340 (60.0%)	0.845
Beta-blockers, *n* (%)	738 (71.9%)	222 (82.2%)	150 (78.9%)	366 (64.6%)	<0.001
Diuretics, *n* (%)	502 (48.9%)	225 (83.3%)	119 (62.6%)	158 (27.9%)	<0.001
spironolactone, *n* (%)	486 (47.3%)	228 (84.4%)	120 (63.2%)	138 (24.3%)	<0.001
Aortic dilation, *n* (%)	313 (30.5%)	79 (29.3%)	54 (28.4%)	180 (31.7%)	0.607

BMI, body mass index; SBP, systolic blood pressure; DBP, diastolic blood pressure; PP, pulse pressure; AoD, aortic diameter; HR, heart rate; WBC, white blood cell; Hb, hemoglobin; HCT, hematocrit; TB, total bilirubin; ALB, albumin; ALT, alanine transaminase; TC, total cholesterol; TG, total triglyceride; LDL-C, low-density lipoprotein cholesterol; HDL-C, high-density lipoprotein cholesterol; eGFR, estimated glomerular filtration rate;UA, uric acid; FBG, fasting blood glucose; LVED, left ventricular end diastolic diameter; LAD, left atrial diameter; DM, diabetes mellitus; CHD, coronary heart disease; DCM, dilated cardiomyopathy; CKD, chronic kidney disease; ACEI, angiotensin converting enzyme inhibitor; ARB, angiotensin receptor blockers; NYHA, New York heart association.

### 3.2 Correlation between PP and AoD in patients with HFrEF

**Figure 1:**
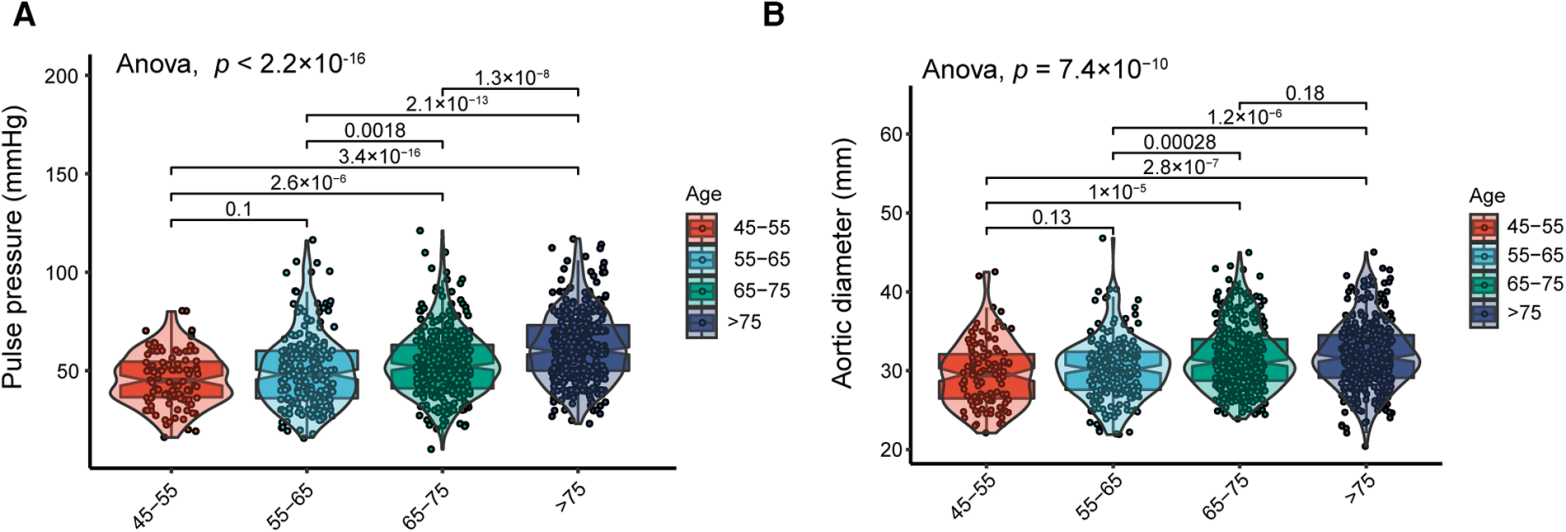
(**A**) Distribution of PP in different age groups; PP increases with age. (**B**) Distribution of AoD in different age groups; AoD increases with advanced age.

**Figure 2:**
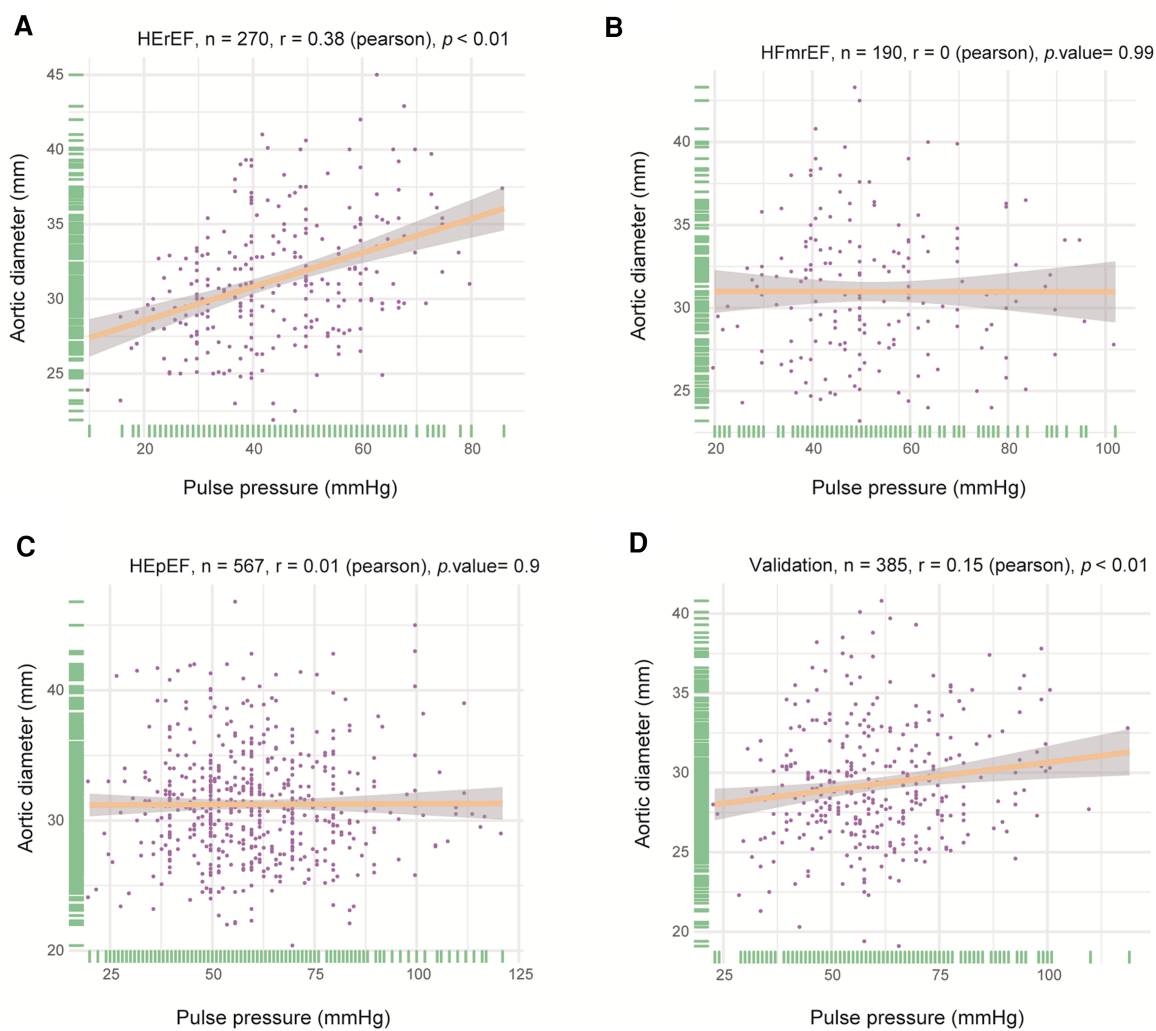
Correlation between PP and AoD in different types of HF. (**A**) PP was positively associated with AoD in HFrEF (*r* = 0.380, *p *< 0.01). (**B**) There was no significant correlation between PP and AoD in HFmrEF. (**C**) There was no significant correlation between PP and AoD in HFpEF. (**D**) PP was positively associated with AoD in validation patients with HFrEF (*r* = 0.15, *p* < 0.01).

PP and AoD were found to increase with age in patients with HF (Figure 1, Supplementary Figure S1). Therefore, we further explored the association between PP and AoD in patients with HFrEF, HFmrEF, and HFpEF. The results showed that PP was positively associated with AoD in the middle-aged and the elderly with HFrEF (*r* = 0.380, *p* < 0.01), but not in patients with HFmrEF (*r* = 0, *p *= 0.99) or HFpEF (*r* = 0.01, *p *= 0.9) (Figure 2).

The independent association of AoD/BSA with other variables was assessed using multiple linear regression analysis. We observed that PP was independently associated with AoD/BSA (*β* = 0.170, *p* < 0.004), adjusting for age, BMI, SBP, DBP, HR, hemoglobin level, triglyceride (TG), total cholesterol(TC), high-density lipoprotein cholesterol (HDL-C), low-density lipoprotein cholesterol (LDL-C), eGFR, LVEF, left ventricular end diastolic diameter (LVED. The independent determinants of AoD/BSA in the fully adjusted models included PP, age, BMI, and ALB (Table 2).

**Table 2:** Multivariate linear regression analysis for AoD and AoD/BSA.

Variables	AoD		AoD/BSA	
	*β*	*p*	β	*p*
Age	0.275	<0.001	0.289	<0.001
BMI	0.179	0.001	−0.323	<0.001
SBP	0.092	0.248	0.048	0.541
DBP	0.063	0.248	0.033	0.541
PP	0.246	<0.001	0.170	0.004
HR	0.002	0.964	0.061	0.254
Hb	0.107	0.057	0.102	0.067
TG	−0.017	0.752	−0.020	0.713
TC	0.019	0.726	0.074	0.175
HDL-C	−0.063	0.259	0.007	0.902
LDL-C	0.061	0.270	0.098	0.074
eGFR	0.095	0.079	0.082	0.124
LVEF	−0.046	0.410	−0.041	0.452
LVED	0.069	0.209	−0.084	0.119

Adjusting variables included age, BMI, blood test indicators, hemodynamic parameters and echocardiographic indicators. AoD, aortic diameter; BSA, body surface area; BMI, body mass index; PP, pulse pressure; SBP, systolic blood pressure; DBP, diastolic blood pressure; HR, heart rate; Hb, hemoglobin; TG, total triglyceride; TC, total cholesterol; HDL-C, high-density lipoprotein cholesterol; LDL-C, low-density lipoprotein cholesterol; eGFR, estimated glomerular filtration rate; LVEF, left ventricular ejection fraction; LVED, left ventricular end diastolic diameter.

**Figure 3:**
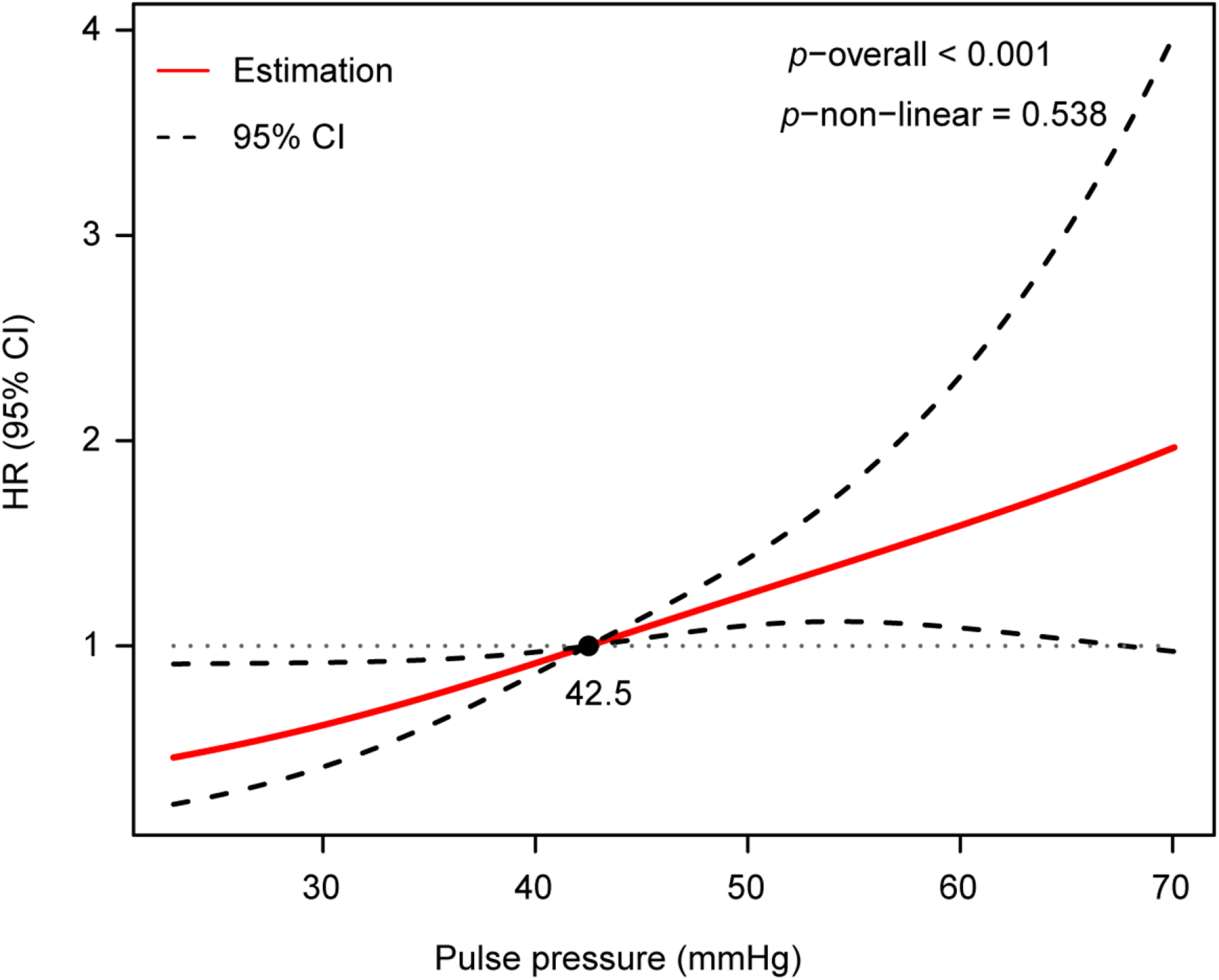
There was no non-linear association between PP and AoD assessed by restricted cubic spline analysis in patients with HFrEF (*p*−non−linear = 0.538, *p*−overall < 0.001).

To assess the non-linear association between PP and AoD, a restricted cubic spline analysis was performed. The results indicated that there was no non-linear association between PP and AoD in patients with HFrEF (Figure 3).

### 3.3 Identification of independent predictors of AoD in patients with HFrEF

The clinical characteristics of the 270 patients with HFrEF based on the quartiles of PP levels are shown in Table 1. The mean age of the patients was 65.7 ± 10.3 years, 201 (74.4%) were males, and 69 (25.6%) were females. The average PP level was 44.8 ± 14.0 mmHg in patients with HFrEF; the lowest quartile (Q1) was 30.8 ± 6.0 mmHg, the second quartile (Q2) was 44.9 ± 4.1 mmHg, third quartile (Q3) was 58.3 ± 3.5 mmHg, and the highest quartile (Q4) was 70.8 ± 5.3 mmHg. The patients in the higher quartile levels of PP were older: 62.6 ± 9.3 (Q1), 66.0 ± 11.0 (Q2), 70.0 ± 9.2 (Q3), and 72.5 ± 9.6 (Q4) years (*p* < 0.001). With ascending quartile levels of PP, a trend of increasing AoD was observed: 30.07 ± 3.57 (Q1), 32.05 ± 4.54 (Q2), 32.22 ± 4.12 (Q3), and 34.68 ± 4.06 mm (Q4) (*p* < 0.001). The SBP was higher in Q4 (151.0 ± 12.3 mmHg) than in Q1 (108.6 ± 15.8 mmHg) (*p *< 0.016). The prevalence of hypertension (17 [73.9%] vs. 43 [33.3%]) and CHD (18 [78.3%] vs. 46 [35.7%]) was higher in Q4 than in Q1 (*p* < 0.016). No significant differences were found in DBP, blood lipids, percentage of diabetes, and use of medications between groups (Table 3).

**Table 3:** Comparison of clinical characteristics of patients with hFrEF in the quartile of PP levels.

Variables	Quartile 1	Quartile 2	Quartile 3	Quartile 4	*F/χ^2^*	*p*
	<40 mmHg	40–52 mmHg	52–64 mmHg	≥65 mmHg		
PP, mmHg	30.8 ± 6.0	44.9 ± 4.1*	58.3 ± 3.5*	70.8 ± 5.3*	641.600	<0.001
AoD, mm	30.07 ± 3.57*	32.05 ± 4.54*	32.22 ± 4.12*	34.68 ± 4.06*	11.378	<0.001
Age, years	62.6 ± 9.3	66.0 ± 11.0	70.0 ± 9.2*	72.5 ± 9.6*	11.637	<0.001
BMI, kg/m^2^	23.03 ± 3.29	24.47 ± 4.49*	24.74 ± 2.74*	24.25 ± 2.74	3.772	0.011
SBP, mmHg	108.6 ± 15.8	125.6 ± 13.7*	135.5 ± 14.1*	151.0 ± 12.3*	82.205	<0.001
DBP, mmHg	75.6 ± 14.6	78.2 ± 13.4	76.6 ± 13.6	79.7 ± 11.3	0.886	0.449
HR, bpm	81.9 ± 20.6	78.5 ± 15.8	81.3 ± 18.6	80.8 ± 18.8	0.483	0.695
TG, mmol/l	1.45 ± 1.47	1.44 ± 1.01	1.48 ± 1.02	1.30 ± 0.57	0.132	0.941
TC, mmol/l	3.98 ± 1.04	4.03 ± 1.30	3.98 ± 1.01	3.96 ± 1.22	0.040	0.989
HDL-C, mmol/l	0.99 ± 0.32	1.07 ± 0.37	1.00 ± 0.33	0.91 ± 0.30	1.664	0.175
LDL-C, mmol/l	2.61 ± 0.93	2.62 ± 1.02	2.46 ± 0.80	2.68 ± 1.16	0.444	0.721
LVED, mm	65.36 ± 11.17	62.23 ± 6.28	63.86 ± 8.21	61.66 ± 8.22	2.136	0.096
LAD, mm	46.54 ± 9.68	45.57 ± 7.33	46.03 ± 9.80	46.73 ± 7.08	0.200	0.896
LVEF, %	29.19 ± 6.73	30.47 ± 5.78	31.79 ± 5.65	32.95 ± 5.58	3.796	0.011
Hypertension, *n* (%)	43 (33.3%)	35 (54.7%)*	36 (66.7%)*	17 (73.9%)*	25.942	<0.001
DM, *n* (%)	41 (31.8%)	20 (31.3%)	23 (42.6%)	12 (52.2%)	5.258	0.154
CHD, *n* (%)	46 (35.7%)	34 (53.1%)*	28 (51.9%)*	18 (78.3%)*	17.161	0.001
ACEI or ARB, *n* (%)	70 (54.3%)	45 (70.3%)	36 (66.7%)	14 (60.9%)	5.527	0.137
Beta-blockers, *n* (%)	109 (84.5%)	51 (79.7%)	43 (79.6%)	19 (82.6%)	0.988	0.804
Diuretics, *n* (%)	111 (86.0%)	53 (82.8%)	43 (79.6%)	18 (78.3%)	1.656	0.647
Spironolactone, *n* (%)	88 (88.9%)	75 (82.4%)	47 (85.5%)	18 (72.0%)	4.763	0.190

Data are expressed as mean ± standard or median (25th–75th quartile).

BMI, body mass index; SBP, systolic blood pressure; DBP, diastolic blood pressure; PP, pulse pressure; AoD, aortic diameter; HR, heart rate; TC, total cholesterol; TG, total triglyceride; LDL-C, low-density lipoprotein cholesterol; HDL-C, high-density lipoprotein cholesterol; LVED, left ventricular end diastolic diameter; LAD, left atrial diameter; LVEF, left ventricular ejection fraction; DM, diabetes mellitus; CHD, coronary heart disease; DCM, dilated cardiomyopathy.

* *p *< 0.016 vs. quartile 1.

Multiple regression analysis was employed to assess the association between PP levels and aortic root dilation in three models. In the univariate logistic regression analysis, with Q1 set as the reference, the PP levels in Q4 were associated with an increased odds ratio (OR) for aortic root dilation [OR = 5.685 95% confidence interval (CI): 1.941–16.646, *p* for trend = 0.015]. After adjusting for age, sex, BSA, smoking, hypertension, we observed that the PP levels in Q4 were associated with an increased OR for aortic root dilation (OR = 4.897, 95% CI: 1.629–14.717, *p* for trend = 0.043) as compared with the PP levels in Q1. Moreover, when adjusting for complications and hematological markers simultaneously, those in Q4 had a higher risk of aortic root dilation than those in Q1 (Q4: 6.612, 95% CI: 1.838–23.783 vs. Q1: 2.411, 95% CI: 1.037–5.607; *p* for trend = 0.049) (Table 4). Subsequently, stratified analysis was performed to explore further the association between PP and AoD in different population settings, including age, BMI, hypertension, LDL-C, and smoking. An interaction test was performed to assess any significant dependence of the effect modifier on this association. We observed a significant modification of the association between PP levels and AoD (*p* for interaction = 0.047). A significant association between PP levels and AoD was only observed in patients ≥ 65 years old (OR = 1.753, 95% CI: 1.159–2.652, *p* for trend = 0.008), but not in patients < 65 years old (*p* for trend = 0.930). No significant interaction was observed in BMI, LDL-C, smoking status, and hypertension (*p* for interaction = 0.540, 0.571, 0.470, and 0.289, respectively), indicating that the magnitude of the relationship was the same for different population settings concerning these variables (Table 5).

**Table 4:** The association between PP levels and aortic dilation in HFrEF.

	Quartile of PP				
	Q1 (<40 mmHg)	Q2 (40–52 mmHg)	Q3 (52–64 mmHg)	Q4 (≥65 mmHg)	*p*_trend_
	OR (95% CI)	OR (95% CI)	OR (95% CI)	OR (95% CI)	
Model 1	1	2.697 (1.275–5.703)	1.471 (0.613–3.526)	5.685 (1.941–16.646)	0.015
*p* values		0.009	0.387	0.002	
Model 2	1	2.570 (1.204–5.485)	1.280 (0.525–3.125)	4.897 (1.629–14.717)	0.043
*p* values		0.015	0.587	0.005	
Model 3	1	2.411 (1.037–5.607)	1.163 (0.431–3.138)	6.612 (1.838–23.783)	0.049
*p* values		0.041	0.766	0.004	

Model 1 was adjusted for age, pulse pressure, gender, and body surface area.

Model 2 was adjusted for covariates in model 1 plus smoking status, hypertension.

Model 3 was adjusted for covariates in model 2 plus cardiac index (left atrial diameter and ejection fraction), hematological index, diabetes mellitus, coronary heart disease, chronic kidney disease, and stroke.

**Table 5:** Stratified association of PP levels and increased aortic dilation in HFrEF.

	PP quartiles (mmHg)	
	OR (95% CI), *p* for trend	*p* for interaction
Age(years)		
<65	0.971 (0.499–1.887), 0.930	0.047
≥65	1.753 (1.159–2.652), 0.008	
BMI (kg/m^2^)		
≥24	1.418 (0.913–2.201), 0.120	0.540
<24	1.455 (0.857–2.470), 0.165	
Hypertension		
No	1.144 (0.646–2.028), 0.644	0.289
Yes	1.719 (1.135–2.605), 0.011	
LDL-C (mmol/l)		
<3.3 mmol/l	1.294 (0.889–1.882), 0.178	0.571
≥3.3 mmol/l	3.203 (1.211–8.475), 3.203	
Past smoking		
No	1.531 (0.962–2.436), 0.072	0.470
Yes	1.387 (0.874–2.202), 0.165	

PP quartiles (mmHg): Q1: <40 mmHg Q2: 40–52 mmHg Q3: 52–64 mmHg Q4: ≥65 mmHg.

Adjusted for sex, HDL-C, LVEF, LVED, past drinking and age, BMI, LDL-C, past drinking, and hypertension when they were not the strata variables. BMI, body mass index; PP, pulse pressure; LDL-C, low-density lipoprotein cholesterol; HDL-C, high-density lipoprotein cholesterol; LVED, left ventricular end diastolic diameter; LVEF, left ventricular ejection fraction.

### 3.4 Validation dataset for relationship of PP and AoD

Based on the discovery data, we validated the relationship between PP and AoD in another dataset. The information for the validation data is shown in Supplementary Table S1. Then, we further validated the association between PP and AoD in patients with HFrEF. The results showed that PP was positively associated with AoD in patients with HFrEF (*r* = 0.15, *p* < 0.01; Figure 2D). The correlations were further assessed by linear regression analysis. We observed that PP was independently associated with AoD (*β* = 0.205, *p* < 0.001), adjusting for age, BMI, blood test indicators, hemodynamic parameters and echocardiographic indicators (Supplementary Table S2).

## 4 Discussion

In our study, age was lowest in HFrEF group compared to the other kinds of heart failure. Although the management of HFrEF has seen significant scientific breakthrough in recent decades, HFrEF is a major public health concern with substantial morbidity and mortality. Disease morbidity and mortality remain high, with a 5-year survival rate of 25% after hospitalization for HFrEF (32). Among the three groups of heart failure types, the LVED of HFrEF was larger than that of the other two groups, suggesting that HErEF was more likely to undergo ventricular remodeling. Deleterious LV remodeling, including increases in end-diastolic and end-systolic volume and reduction in LVEF, is pathognomonic of HFrEF, and the extent of adverse remodeling correlates with risk of hospitalization and death. LV volumes and contractility worsen progressively over time (33). This also suggests that early intervention should be initiated for the treatment of HFrEF. We found that the average level of PP was 44.8 ± 14.0 mmHg in patients with HFrEF, 52.6 ± 16.5 mmHg in patients with HFmrEF, and 60.3 ± 17.5 mmHg in patients with HFpEF in our study. These findings are similar to those of a previous study showing the lowest average level of PP in the HFrEF group (34). This phenomenon may be related to the wide use of angiotensin converting enzyme inhibitors/angiotensin receptor blockers, beta-blockers, and spironolactone in patients with HFrEF, which are known to lower SBP and PP (35). Meanwhile, we found that the systolic blood pressure in the HFpEF group was the highest and their heart rate was the lowest compared to other groups. In contrast, the aortic root diameter was not significantly different between HF classification. Studies (36, 37) showed that the lower SBP being a dose-dependent marker of impaired left ventricular contractility. Advanced heart failure is usually associated with low systolic blood pressure (SBP). Studies (38, 39) have been reported that increased resting heart rate is a risk factor for cardiovascular mortality in cardiovascular diseases. In heart failure patients, increased heart rate has been correlated with adverse outcomes, independently of traditional risk factors. Accordingly, heart rate reduction has been identified as an effective therapy for patients with HF. The aortic root diameter is affected by a variety of factors, including age, gender, height, weight, body surface area (BSA), body mass index (BMI), and diseases such as hypertension, valvular heart disease, congenital heart disease, cardiomyopathy and ischemic cardiomyopathy. In HFrEF group, the aortic root diameter will increase due to cardiac remodeling. However, the aortic root diameter also increases with age. In this study, the age in HFrEF group was smaller, so AoD was significantly different between HF classification because age plays a key role in it. Moreover, we found that PP and AoD increased with age in patients with HF. The aorta has a significant influence on left ventricular afterload and hemodynamics. Age-related enlargement of AoD is a characteristic of arterial stiffness that can influence cardiovascular disease (15, 22, 23). PP, an indicator of pulsatile flow ejected by the heart, is mainly influenced by LVEF and arterial stiffness (40). It has been reported that high PP is usually related to increased SBP and systolic hypertension accelerates arterial stiffness, thereby resulting in CVD (4, 41–43). Epidemiologic studies have shown that PP is predictive of the incidence of HF in the elderly population (44). Several epidemiological studies have examined the relationship between the proximal aorta and major adverse cardiovascular events. For example, the Cardiovascular Health Study (CHS) (45) and the study by Cuspidi et al. (46) showed that increased AoD was associated with a higher incidence of cardiovascular events. However, the relationship between PP and AoD remains controversial. Cuspidi et al. (24) found a positive association between PP and AoD (*r* = 0.10, *p* = 0.004) in a total of 3,366 treated and untreated patients with essential hypertension. In a community-based cohort (*n* = 3,108), Daisuke et al. (22) observed that participants in the highest AoD quintile (35.2 ± 2.8 mm) had higher PP than those in the lowest quintile (30.8 ± 2.8 mm). Recently, a study in Korea demonstrated a significant positive association between invasively measured aPP and AoD/BSA (47). In contrast, there was an independent inverse relationship between AoD and PP in the Framingham cohort study (23) and in a study by Agmon et al. (25) The authors considered that the inverse association between AoD and PP might be due to reduced wave reflection as a result of higher aortic compliance in those with larger AoD and considered invasive aortic root PP data as a for possible explanation for this negative association (48). In this study, Pearson correlation analysis showed that PP was positively correlated with AoD in patients with HFrEF. Furthermore, the probability of increased aortic dilation was associated with higher PP quartiles, and a higher risk of aortic dilation was observed in patients with PP ≥ 65 mmHg, which is inconsistent with previously reported results. A possible reason for this discrepancy in the findings between this study and those of the Framingham study is related to the different populations examined. This study focused on the middle-aged and the elderly with HF. In addition, we observed a significant interaction between PP levels and age, indicating that PP and age may have an additive effect on AoD. It is well known that aging leads to arterial stiffness, which makes the arterial wall more susceptible to the harmful effects of PP (49). This phenomenon may explain the positive association of PP levels with AoD observed only in patients ≥ 65 years with HFrEF.

To our knowledge, the present study is the first to show that an increased PP is significantly associated with AoD in patients with HFrEF, but not in patients with HFmrEF or HFpEF. One reason might be that AoD can be influenced by age, height, weight, left ventricular structure (15), and lower the levels of LVED in patients with HFmrEF or HFpEF than that in patients with HFrEF. Appropriate management may be an effective strategy for lowering the risk of aortic dilation in elderly patients with HFrEF. There were no specific interventions focused on PP currently. Therefore, blood pressure management may be beneficial in reducing the risk of aortic dilation in the elderly with HFrEF. To further verify the reliability of our conclusions, we collected the dataset in another hospital to find consistent results from different hospital populations. Finally, the results improved the conclusion reliability.

Certain limitations of this study should be noted. First, this was a limited cross-sectional study of Chinese middle-aged and elderly populations; hence, the results cannot prove a causal relationship between PP and AoD in patients with HFrEF. The generalizability of our findings to the general population or other ethnic groups may be limited. Therefore, prospective studies with larger sample sizes are required to further explore causality. Second, we could not completely account for residual confounders because of unmeasured or unknown variables. For example, medications such as antihypertensive and antidiabetic drugs may affect AoD (50, 51). Additionally, the detailed molecular mechanisms supporting our findings remain elusive; therefore, laboratory-based experiments are required to gain insight into the association between PP and AoD. Finally, due to our lack of measurements, there is no central pulse pressure data. This is a retrospective study, and it is true that the central hemodynamic study data is more revealing and convincing. In fact, the population in our study is mainly about heart failure patients, and the central hemodynamic test is an invasive examination, which is not beneficial to the patients with heart failure, and only a few research institutions can carry out it in clinical practice. The results we obtained can attract the attention of qualified researchers for further validation, so that they can be generalized with simple clinical indicators. Indeed, there is a physiological difference between central artery pressure and peripheral artery pressure, that is, the physiological amplification of PP (52). It is well known that the degree of pulse pressure amplification is strongly associated with total mortality and major cardiovascular events (26).

## 5 Conclusions

Our findings indicated that PP level was independently and positively associated with AoD, especially in older patients with HFrEF, but not in patients with HFmrEF and HFpEF. Arterial stiffening or vascular aging may play a certain role in the older HFrEF patients.

## Data Availability

The original contributions presented in the study are included in the article/Supplementary Material, further inquiries can be directed to the corresponding author.
