# Supplementary material for: Association of pulse pressure and aortic root diameter in elderly Chinese patients with chronic heart failure
Source: Front Cardiovasc Med. 2024 Mar 1;11:1366282. doi: 10.3389/fcvm.2024.1366282 (PMC10940542; doi:10.3389/fcvm.2024.1366282)
Supplement: Supplementary file 1 [file Datasheet1.docx]

**Supplementary Material**

**Supplementary Figure 1**

**
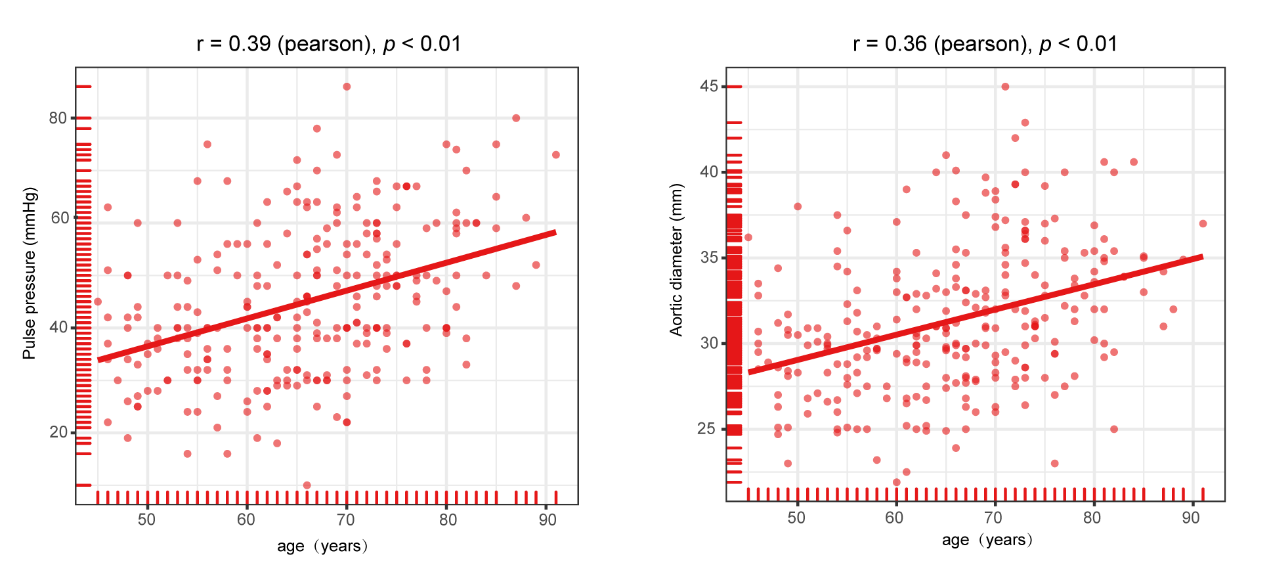
**

**Supplementary Table 1** The clinical characteristics of patients with HFrEF in validation dataset.

|  | **Patients with HFrEF**  **n=378** |
| --- | --- |
| **Age, years** | 70.6 ± 14.6 |
| **Male, n (%)** | 232 (60.3%) |
| **BMI, kg/m^2^** | 23.04 ± 3.44 |
| **SBP, mmHg** | 133.1 ± 17.4 |
| **DBP, mmHg** | 74.2 ± 12.0 |
| **PP, mmHg** | 59.9 ± 16.5 |
| **AO, mm** | 29.20 ± 4.37 |
| **HR, bpm** | 78.9 ± 15.4 |
| **WBC, × 10^9^/L** | 7.67 ± 3.51 |
| **Hb, g/L** | 115.3 ± 29.7 |
| **ALB, g/L** | 36.66 ± 6.22 |
| **ALT(IU/L) (log-transformed)** | 2.98 ± 0.85 |
| **AST(IU/L) (log-transformed)** | 3.25 ± 0.71 |
| **TG, mmol/L** | 1.41 ± 0.98 |
| **TC, mmol/L** | 3.96 ± 1.28 |
| **HDL-C, mmol/L** | 1.05 ± 0.40 |
| **LDL-C, mmol/L** | 2.53 ± 1.08 |
| **eGFR, mL/min/1.73m^2^** | 70.60 ± 39.24 |
| **UA, µmol /L** | 419.0 ± 141.4 |
| **FBG, mmol/L** | 6.30 ± 2.39 |
| **NT-proBNP(pg/ml)**  **(log-transformed)** | 7.59 ± 1.49 |
| **LVED, mm** | 63.20 ± 8.95 |
| **LAD, mm** | 46.40 ± 74.60 |
| **LVEF, %** | 33.8 ± 5.3 |
| **NYHA, n (%)** |  |
| I | 15 (3.9%) |
| II | 138 (35.8%) |
| III | 165 (42.9%) |
| IV | 67 (17.4%) |
| **Hypertension, n (%)** | 290 (75.3%) |
| **DM (n, %)** | 159 (41.3%) |
| **Aortic dilation, n (%)** | 70 (18.2%) |

Abbreviation: BMI, body mass index; SBP, systolic blood pressure; DBP, diastolic blood pressure; PP, pulse pressure; AoD, aortic diameter; HR, heart rate; WBC, [white](javascript:;) [blood](javascript:;) [cell](javascript:;); Hb, [hemoglobin](javascript:;); HCT, hematocrit; TB, [total bilirubin](javascript:;); ALB, albumin; ALT, alanine transaminase; TC, total cholesterol; TG, total triglyceride; LDL-C, low-density lipoprotein cholesterol; HDL-C, high-density lipoprotein cholesterol; eGFR, estimated glomerular filtration rate ;UA, uric acid; FBG, fasting blood glucose; LVED, left ventricular end diastolic diameter; LAD, left atrial diameter; LVEF, left ventricular ejection fraction; NYHA, New York Heart Association.

**Supplementary Table 2 Univariate and stepwise multivariate linear regression analysis for AoD/BSA**

| **Variables** | **Univariate** | | **Multivariate** | |
| --- | --- | --- | --- | --- |
|  | Β | *p* | β | *p* |
| **Age** | -0.036 | 0.634 | -0.086 | 0.180 |
| **BMI** | -0.347 | < 0.001 | -0.355 | < 0.001 |
| **PP** | 0.203 | 0.005 | 0.205 | 0.001 |
| **HR** | 0.062 | 0.374 | 0.070 | 0.272 |
| **WBC** | 0.043 | 0.546 | 0.083 | 0.188 |
| **Hb** | -0.093 | 0.616 | 0.038 | 0.559 |
| **HCT** | 0.142 | 0.437 | 0.055 | 0.401 |
| **ALT** | -0.005 | 0.964 | 0.035 | 0.578 |
| **AST** | 0.011 | 0.918 | 0.017 | 0.784 |
| **TG** | 0.043 | 0.670 | 0.029 | 0.654 |
| **TC** | -0.377 | 0.308 | 0.059 | 0.347 |
| **HDL-C** | 0.050 | 0.697 | -0.054 | 0.395 |
| **LDL-C** | 0.397 | 0.222 | 0.094 | 0.135 |
| **eGFR** | 0.002 | 0.986 | 0.020 | 0.759 |
| **UA** | -0.090 | 0.267 | -0.029 | 0.645 |
| **FBG** | -0.105 | 0.133 | -0.064 | 0.313 |
| **K** | 0.065 | 0.358 | 0.056 | 0.375 |
| **Na** | -0.084 | 0.231 | -0.104 | 0.101 |
| **NT-proBNP** | 0.078 | 0.335 | 0.059 | 0.349 |
| **LVEF** | 0.035 | 0.639 | -0.015 | 0.814 |
| **LVED** | 0.137 | 0.077 | 0.106 | 0.094 |
| **LAD** | -0.057 | 0.428 | -0.004 | 0.945 |

Adjusting variables included age, BMI, blood test indicators, hemodynamic parameters and echocardiographic indicators. Abbreviations: AoD, aortic diameter; BSA, body surface area; BMI, body mass index; PP, pulse pressure; HR, heart rate; WBC, [white](javascript:;) [blood](javascript:;) [cell](javascript:;); Hb, [hemoglobin](javascript:;);HCT, hematocrit; ALT, alanine transaminase; AST, aspartate aminotransferase; TG, total triglyceride; TC, total cholesterol; HDL-C, high-density lipoprotein cholesterol; LDL-C, low-density lipoprotein cholesterol; eGFR, estimated glomerular filtration rate ;UA, uric acid; FBG, fasting blood glucose; K, potassium ion Na, [sodium ion](http://www.baidu.com/link?url=GKoibmZzukTvJtLbFO5JXjucaJ0l5jktPaZWjTYQsFoRTR9Xtt_LjZ08yfeBfnw1IiFBWiuxPjfYc-Rs5zLpyB8FyXFVFaNrjE0176aJ7IS);LVEF, left ventricular ejection fraction; LVED, left ventricular end diastolic diameter; LAD, left atrial diameter.
